# Recent Developments in the Synthesis of HIV-1 Integrase Strand Transfer Inhibitors Incorporating Pyridine Moiety

**DOI:** 10.3390/ijms24119314

**Published:** 2023-05-26

**Authors:** Alexey M. Starosotnikov, Maxim A. Bastrakov

**Affiliations:** N.D. Zelinsky Institute of Organic Chemistry RAS, Leninsky Prosp. 47, 119991 Moscow, Russia; b_max82@mail.ru

**Keywords:** human immunodeficiency virus, HIV-1 integrase, inhibitors, pyridine, synthetic pathways

## Abstract

Human immunodeficiency virus (HIV) causes one of the most dangerous diseases—acquired immunodeficiency syndrome (AIDS). An estimated about 40 million people are currently living with HIV worldwide, most of whom are already on antiretroviral therapy. This makes the development of effective drugs to combat this virus very relevant. Currently, one of the dynamically developing areas of organic and medicinal chemistry is the synthesis and identification of new compounds capable of inhibiting HIV-1 integrase—one of the HIV enzymes. A significant number of studies on this topic are published annually. Many compounds inhibiting integrase incorporate pyridine core. Therefore, this review is an analysis of the literature on the methods for the synthesis of pyridine-containing HIV-1 integrase inhibitors since 2003 to the present.

## 1. Introduction

The human immunodeficiency virus (HIV) causes one of the most fatal diseases—acquired immunodeficiency syndrome (AIDS). The high growth rate in the number of HIV-infected people makes the development of effective drugs to combat this virus very relevant. An extremely important and promising task of modern virology and medicinal chemistry is also the creation of compounds that do not cause the emergence of resistant strains of HIV-1 and/or have inhibitory activity against them. Currently, the main approach to the treatment of HIV infection is antiretroviral therapy (ARVT), which consists of the continuous intake of several antiviral drugs. ARVT can significantly improve the quality and increase the life expectancy of the patient. About 35 drugs are used to treat HIV infection—inhibitors of one of the three HIV enzymes (reverse transcriptase, protease, and integrase). One of the limitations of the use of ARVT is the formation of resistant forms of the virus during therapy, and, therefore, the field of chemistry associated with the synthesis of novel anti-HIV molecules continues to be in demand and relevant.

**Figure 1:**
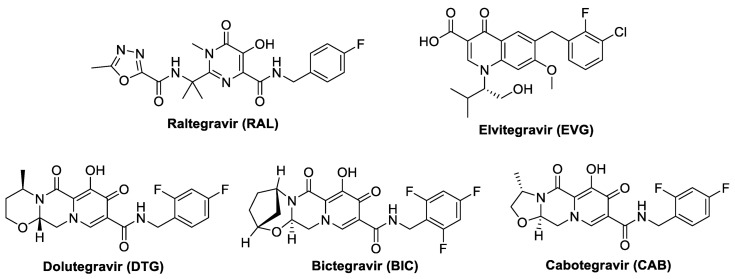
FDA-approved HIV-1 integrase inhibitors.

There are two major types of HIV (HIV-1 and HIV-2), which in turn consist of several groups and subtypes. HIV-1 is more virulent and transmissible than HIV-2, and most of the undertaken efforts have thus been targeted at developing inhibitors of HIV-1 enzymes. In particular, HIV-1 integrase strand transfer inhibitors (INIs) are being designed to block the action of integrase, an enzyme that inserts the viral genome into the DNA of the host cell—one of the most important steps in retroviral replication. To date five HIV-1 integrase inhibitors have been approved: Raltegravir (RAL, approved by the FDA in 2007), Elvitegravir (EVG, 2012), Dolutegravir (DTG, 2013), Bictegravir (BIC, 2018), and Cabotegravir (2021) (see Figure 1). It should be noted that four of them contain a pyridine moiety, thus emphasizing the importance of pyridine derivatives as INIs.

The first INI—Raltegravir was approved for use as a new agent for AIDS therapy in 2007 [1], but it was noticed that in some cases, the virus develops resistance to this new drug within 3 months [2]. The design of novel HIV-1 INIs is based generally on the modification of structure of these five well-established drugs, i.e., the central heterocyclic fragment usually incorporate a pyridine, pyrimidine, or quinoline system. In addition, compounds of some other classes are also being studied, such as indoles, isoindoles, acyclic 2,4-diketoacids, etc. A number of review articles devoted to the current use of INIs as well as discussion of their structural diversity have been published recently [3,4,5,6,7,8,9,10]. At the same time, lack of attention in the review literature has been paid to the chemical synthesis of pyridine-containing INIs. Some information can be found in two publications [11,12]. This review is an analysis of the literature on methods for the synthesis of HIV-1 integrase inhibitors of pyridine and the fused pyridine series covering the period from 2003 to the present. For convenience, the considered compounds of different structures are grouped into separate subsections: monocyclic pyridines, benzoannulated pyridines (quinolines and isoquinolines), and pyridines fused with heterocycles.

## 2. Synthesis of Pyridine-Based HIV-1 Integrase Inhibitors

### 2.1. Monocyclic Pyridines

**Scheme 1:**
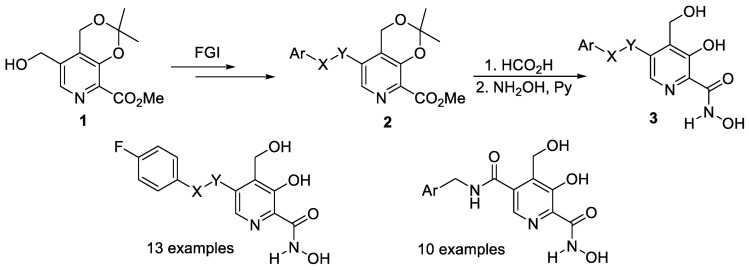
Synthesis of pyridoxine hydroxamic acids.

Most of the publications dealt with HIV-1 INIs over past 20 years are dedicated to development of more efficient syntheses of approved drugs and their structural analogs. Nevertheless, some monocyclic pyridine derivatives have been designed and tested as well. Thus, a new series of pyridoxine hydroxamic acids were synthesized and their antiviral properties were evaluated [13]. The authors studied a large set of pyridines with various spacers X-Y and aryl groups in position 5, Scheme 1. The structural diversity was achieved by common functional group interconversion reactions of 5-hydroxymethyl derivative **1**, such as oxidation to aldehyde or carboxylic acid, conversion of the alcohol to mesylate, or halomethyl derivatives, etc. Cleavage of the acetone protection in **2** and conversion of an ester into hydroxamic acid gave target compounds **3**. It was found that antiviral potency depends on the structure of spacer X-Y and substituents in the aryl group. The best results were observed in case of X − Y = NH-CH_2_; NH-C(O); O-CH_2_ and Ar = 4-F-C_6_H_5_; 3-Cl-4-F-C_6_H_4_; 3,4-Cl_2_-C_6_H_4_.

**Scheme 2:**
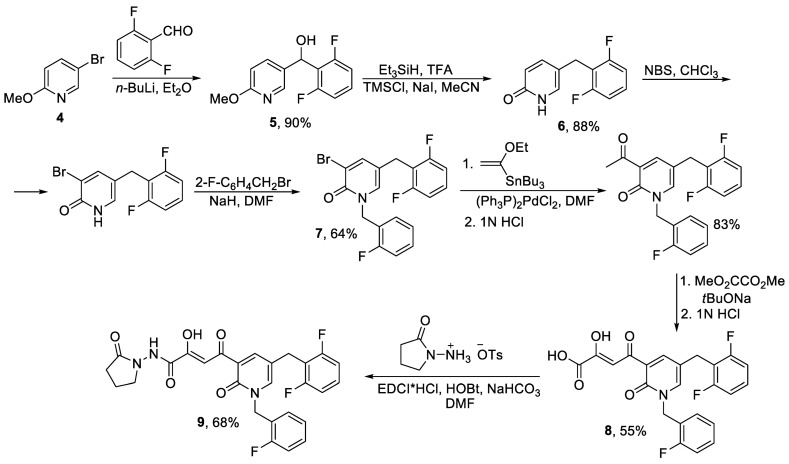
Nair’s synthesis of novel HIV-1 INI **9**.

Nair et al. reported on the discovery of a novel anti-HIV active INI possessing low toxicity and inhibition EC_50_ value of 19-35 nM depending on the HIV subtype [14,15]. The multistep synthesis (Scheme 2) started with readily available pyridine and benzene derivatives and comprised lithiation of 5-bromo-2-methoxypyridine (**4**) followed by reaction with 2,6-difluorobenzaldehyde. Dehydroxylation and cleavage of methoxy protection in compound **5** afforded pyridine-2-one derivative **6** in 88% yield. Bromination and N-alkylation of a pyridine moiety gave an intermediate product **7** in 64% yield over two steps. The introduction of the acetyl group was achieved using Pd-catalyzed Stille cross-coupling with 1-ethoxyvinyl(tributyl)stannane followed by acidic work-up. Transformation of the acetyl group into diketoacid derivative **8** was performed in the usual manner via reaction with diethyl oxalate and successive acid-catalyzed hydrolysis. Finally, reaction of the acid **8** with 1-amino-2-pyrollidinone *p*-toluenesulfonate led to the target compound **9** with 68% yield.

A similar synthetic scheme was applied for the preparation of β-diketo acid derivatives with 2,4-difluorobenzyl substituent in position 5 [16] and many other diversely substituted 1,5-dibenzylpyridinones [17].

**Scheme 3:**
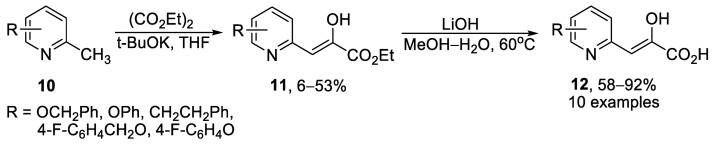
Synthesis of 2-hydroxy-3-pyridylacrylic acid derivatives.

2-Hydroxy-3-pyridylacrylic acid derivatives as novel HIV integrase inhibitors were synthesized on the basis of substituted 2-methylpyridines [18]. Reactions of compounds **10** with diethyl oxalate afforded 2-hydroxy-3-pyridylacrylic esters **11,** which were hydrolyzed under the action of LiOH to give the corresponding acids **12** in moderate to high yields (Scheme 3). It was demonstrated that the position of substituent R in the hydrophobic domain as well as the nature of R provided useful information for the design of novel INI types.

**Scheme 4:**
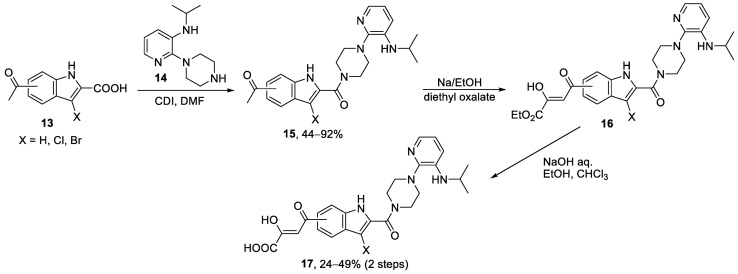
Preparation of pyridine-containing dual inhibitors of HIV reverse transcriptase and integrase.

Dual inhibitors of HIV reverse transcriptase and integrase were synthesized on the basis of pyridine-containing reverse transcriptase inhibitor Delavirdine [19]. The synthesis was accomplished starting with reactions of diversely substituted acetylindole-2-carboxylic acids **13** and 3-isopropylamino-2-(piperazyn-1-yl)-pyridine (**14**) in the presence of carbonyl diimidazole (CDI) to give corresponding amides **15** (Scheme 4). Further reactions with diethyl oxalate and basic hydrolysis of the esters **16** afforded target 2,4-diketoacids **17**. Compounds bearing diketoacid functionality in position 5 of the indole system showed good activity against both enzymes and HIV in cell-based assays, while C-7 isomers were inactive.

**Scheme 5:**
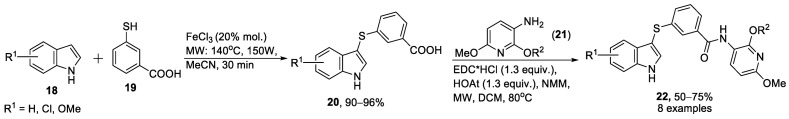
Synthesis of indole-pyridine hybrids as potent HIV-1 INIs.

Design, synthesis, and evaluation of novel small molecules inhibiting an interaction of HIV-1 integrase and Lens epithelium derived growth factor (LEDGF/p75) was reported by Van der Eycken et al. [20]. The substituted indoles **18** were sulfenylated with 3-mercaptobenzoic acid **19** under microwave irradiation conditions to give 3-arylthio- derivatives **20** in high yields (Scheme 5). Reactions of the latter with alkoxyaminopyridines **21** in the presence of EDC hydrochloride and 1-hydroxy-7-azabenzotriazole (HOAt), also under microwave irradiation, afforded target amides **22** in moderate isolated yields. The inhibiting activity was found to be dependent on both R^1^ and R^2^ substituents: 5-chloroindoles were inactive, while 2-isopropyloxypyridine derivatives demonstrated higher activity in comparison with 2-methoxy and 2-butyloxy compounds.

**Scheme 6:**
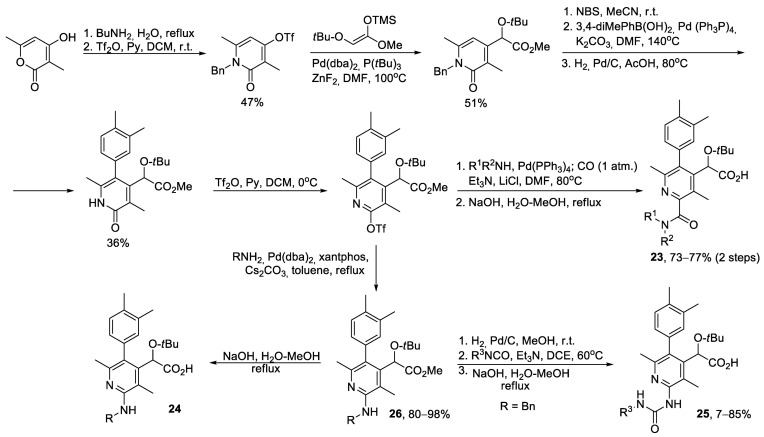
Sugiyama’s approach to novel integrase-LEDGF/p75 allosteric inhibitors.

Novel integrase-LEDGF/p75 allosteric inhibitors based on pyridine scaffold were discovered by Sugiyama and coworkers, Scheme 6. [21]. This multistep synthesis provided a series of 5-aryl-3,6-dimethylpyridines **23**–**25** with various functions in position 2 on the basis of simple pyran-2-one. The authors successfully used Pd-catalyzed cross-coupling reactions to introduce aryl and amine fragments via corresponding trifluoromethanesulfonates. Substituents with intramolecular hydrogen bond at C-2, such as urea derivatives **25**, are desirable for increasing antiviral activity. These compounds were obtained from 2-aminopyridines **26** by the reaction with isocyanates.

**Scheme 7:**
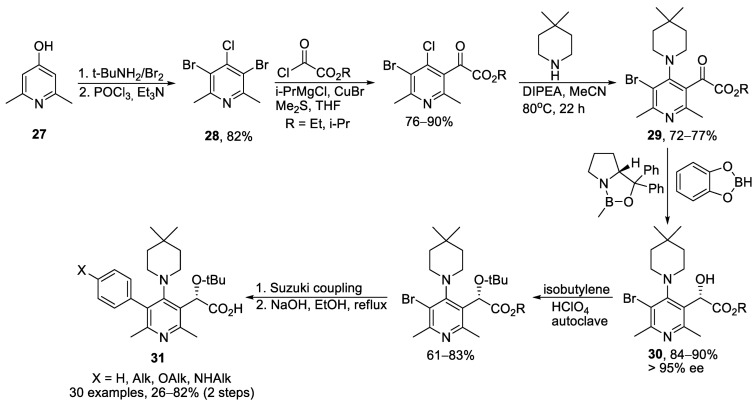
Synthetic route to pyridine-based allosteric INIs **31**.

A series of pyridine-based allosteric INIs have been synthesized recently by Naidu et al. [22]. The authors proposed an elegant reaction sequence starting with 4-hydroxy-2,6-dimethylpyridine (**27**) (Scheme 7). It was subjected to dibromination followed by the conversion of hydroxyl to chlorine under the action of POCl_3_ to give intermediate **28**. One of the bromine atoms was then replaced with 1,2-dicarbonyl fragment, and 4-Cl was substituted with 4,4-dimethylpiperidine. Further asymmetric reduction of the carbonyl group in compound **29** gave the corresponding alcohols **30,** which, after protection, were involved in Suzuki–Miyaura cross-coupling with various arylboronic acids and ester hydrolysis. Finally, some functional group interconversions were made to extend the raw of target compounds **31**. One of the synthesized compounds (X = 4-F-C_6_H_4_CH_2_CH_2_O) was selected as the preclinical lead based on its promising antiviral potency, but multidose toxicity studies in rats revealed adverse results, which caused discontinuation of the further development.

**Scheme 8:**
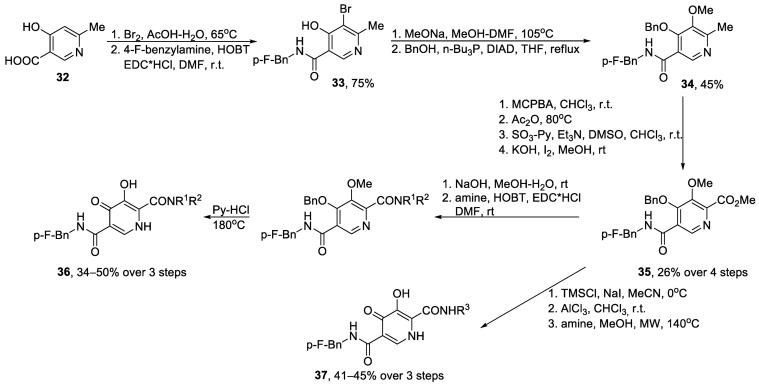
Synthesis of carbamoylpyridine-4-ones—novel HIV-1 INIs.

Carbamoyl pyridone chelating scaffold was designed in 2012 [23]. It was considered as an advanced two-metal binding pharmacophore that demonstrates high activity in both enzymatic and antiviral assay formats. 4-Hydroxy-6-methylnicotinic acid (**32**) was brominated and converted to 4-F-benzylamide **33** using HOBT and EDC (Scheme 8). The Bromine atom was then replaced with NaOMe and the benzyloxy group was introduced through a Mitsunobu reaction with benzyl alcohol, yielding compound **34**. The 2-Methyl group of **34** was converted to methoxycarbonyl via subsequent formation of pyridine-N-oxide, its reaction with Ac_2_O to give 2-hydroxymethyl compound, its oxidation to aldehyde, and further to methyl ester. Thus, the key intermediate **35** was synthesized and used for the preparation of various primary and secondary amides **36** and **37**. Although none of the synthesized compounds showed necessary potency against key resistant mutants, the authors positioned their study as an attractive starting point for further research. Some other 3-hydroxy-5-carbamoylpyridin-4-ones were patented as HIV-1 INIs [24].

**Scheme 9:**
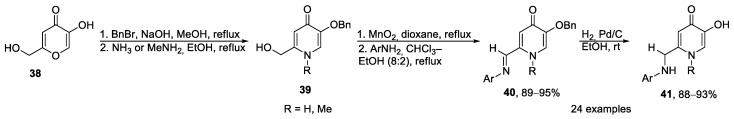
Approach to 3-hydroxypyridine-4-ones.

Substituted 3-hydroxypyridine-4-ones were synthesized on the basis of kojic acid and tested as potential HIV-1 INIs, Scheme 9 [25]. The reaction of kojic acid (**38**) with BnBr in basic media followed by interaction with amine afforded 2-hydroxymethylpyridin-4-ones **39**, which then were oxidized to the corresponding aldehydes using MnO_2_ and condensed with arylamines to give Shiff bases **40**. On hydrogenation of the latter, the benzyl group was cleaved, accompanied by the reduction of the C=N double bond. The Shiff bases **40** exhibited higher anti-HIV activities than their hydrogenation products **41**—IC_50_ 65–100 μM and 90–1000 μM, respectively.

**Scheme 10:**
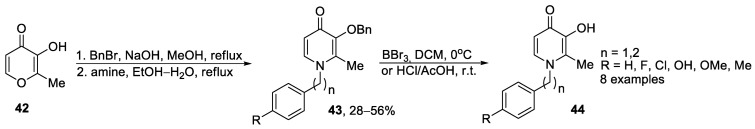
Synthesis of 3-hydroxypyridin-4-ones **44**.

Another example of 3-hydroxypyridin-4-one synthesis was reported by Sirous et al., Scheme 10 [26]. 3-Hydroxy group of the commercially available maltol (**42**) was protected by the reaction with benzyl bromide and further reactions with benzyl- or phenetylamines gave 1-substituted pyridine-4-ones **43**. Cleavage of the benzyl group was carried out using boron tribromide or a mixture of hydrochloric and acetic acids. The target 3-OH derivatives **44** demonstrated lower activities with respect to the correspondingly substituted pyran-4-one compounds, which represent a valuable scaffold for developing efficient INIs.

### 2.2. Quinolines and Isoquinolines

**Scheme 11:**
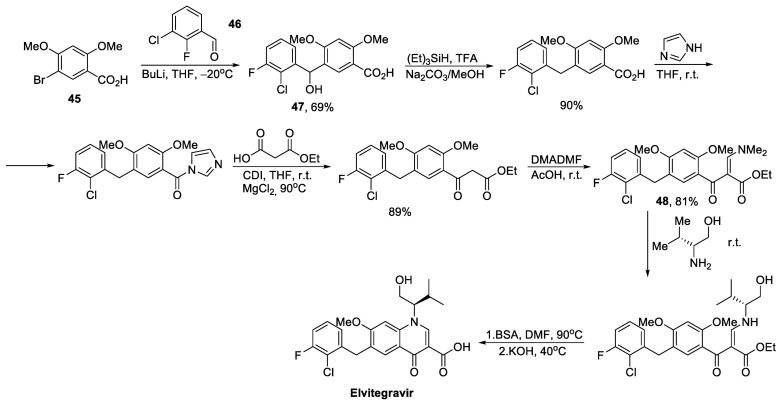
Original synthesis of Elvitegravir.

Elvitegravir (EVG, GS9137) is one of the famous quinoline containing drugs used in anti-HIV 1 therapy. It was developed by the Gilead Sciences, which in 2008 licensed EVG from Japan Tobacco [27]. The synthetic route for the preparation of this compound was described in patent [28] (Scheme 11). It comprised the reaction of 5-bromo-2,4-dimethoxybenzoic acid (**45**) with 2-chloro-3-fluorobenzaldehyde (**46**) followed by dehydroxylation and formation of imidazolide **47**. In the next step, β-ketoester functionality was introduced and reaction with DMADMF afforded the corresponding enamine **48**. The quinoline skeleton was constructed in a further 3 steps: reaction with L-valinol, *N,O*-bis-(trimethylsilyl)acetamide, and potassium hydroxide.

The invention of Elvitegravir stimulated the development of synthetic approaches, leading to structurally similar quinoline compounds and the study of their activity toward HIV-1 integrase.

**Scheme 12:**
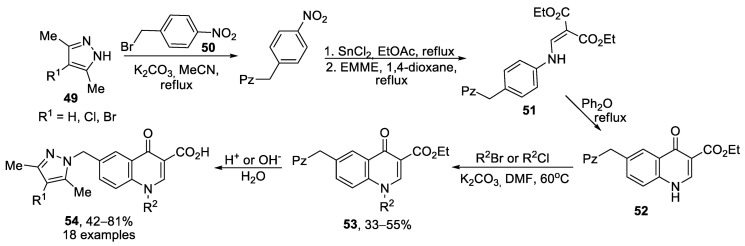
Hu’s synthesis of pyrazolyl-4-oxo-4*H*-quinoline-3-carboxylic acids **54**.

A series of novel pyrazolyl-4-oxo-4*H*-quinoline-3-carboxylic acids bearing various substituents on the N-position of quinoline ring that are structural analogs of EVG were designed and synthesized by Hu et al. [29]. 4-Substituted 3,5-dimethylpyrazoles **49** react with *p*-NO_2_-benzyl bromide (**50**) followed by reduction of the nitro group and the introduction of methylenemalonate fragment, giving rise to the corresponding enamines **51**, (Scheme 12), which on heating in diphenyl ether gave 4-quinolone compounds **52**. Quinolones **52** were N-alkylated by various alkyl halides to give derivatives **53**, and after basic or acidic hydrolysis, the target compounds **54** were obtained. However, shuffling of pharamacophore fragments at N(1) and R^1^ did not lead to any obvious inhibitory activity.

**Scheme 13:**
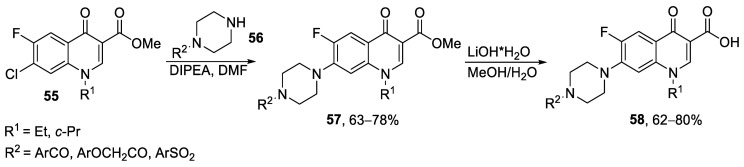
Synthesis of 6-fluoro-4-quinolone-3-carboxylic acids as potential HIV-1 INIs.

Patil et al. reported on docking and synthesis of 6-fluoro-4-quinolone-3-carboxylic acids as potential HIV-1 INIs [30,31]. The target compounds synthesized via simple two-step procedure (Scheme 13). Reaction of 7-chloro-6-fluoroquinolone-3-carboxylates **55** with *N*-substituted piperazines **56** afforded chlorine-substitution products **57** in moderate yields. Hydrolysis of ester bonds with LiOH gave target compounds **58**, which were evaluated for their enzymatic activity against HIV-1. Some of the synthesized compounds exhibited moderate to good anti-HIV-1 integrase inhibitory activity in comparison with the reference drugs.

**Scheme 14:**
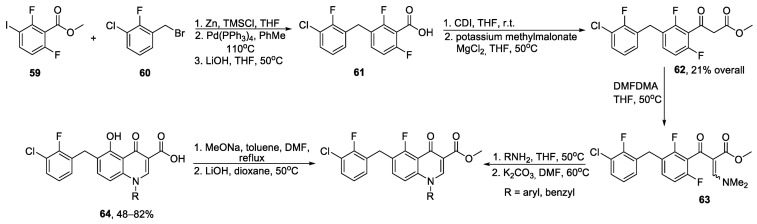
Synthesis of novel 5-R-quinolone carboxylic acids—structural analogs of Elvitegravir.

Chen’s group reported on the synthesis of some novel 5-R-quinolone carboxylic acids—structural analogs of Elvitegravir [32,33]. The target compounds were obtained according to classical synthetic route for assembling quinoline skeleton as outlined on Scheme 14. Negishi coupling of methyl 2,6-difluoro-3-iodobenzoate (**59**) and 3-chloro-2-fluorobenzyl bromide (**60**) followed by ester hydrolysis afforded highly functionalized intermediate **61**. Carboxylic acid was then converted to β-ketoester **62** and reacted with DMADMF to give the enamine **63**—a precursor of the quinoline ring system. The pyridine ring was annulated after reaction with aryl- or benzylamine and treatment with K_2_CO_3_. All synthesized compounds **64** showed significant inhibition activity in low micromolar concentration range. In addition, the author revealed influence of N-substituent on inhibition (IC_50_) and antiviral activity (EC_50_). Some other structurally similar 6-benzylquinolone-3-carboxylic acids were reported in [34].

**Scheme 15:**
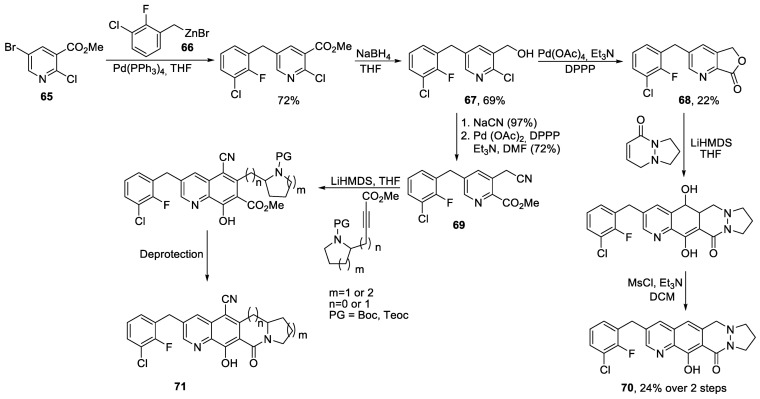
Preparation of 8-hydroxyquinoline tetracyclic lactams.

Velthuisen et al. designed 8-hydroxyquinoline tetracyclic lactams as HIV-1 INIs [35]. The authors described a method for the synthesis of quinolines via benzene ring annulation (Scheme 15). Negishi coupling of the commercially available methyl 5-bromo-2-chloronicotinate (**65**) with (3-chloro-2-fluorobenzyl)zinc bromide (**66**), followed by the reduction afforded 2-chloro-3-hydroxymethyl pyridine **67**—a key intermediate in the proposed synthetic scheme. Compound **67** and its 3-cyanomethyl analog were carbonylated to give esters **68** and **69**, respectively, which were converted to tetracyclic lactams **70** and **71** over two additional steps. Some of the synthesized compounds have exceptional antiviral activity against HIV-1 and virological profile consistent with other second generation integrase stand transfer inhibitors.

**Scheme 16:**
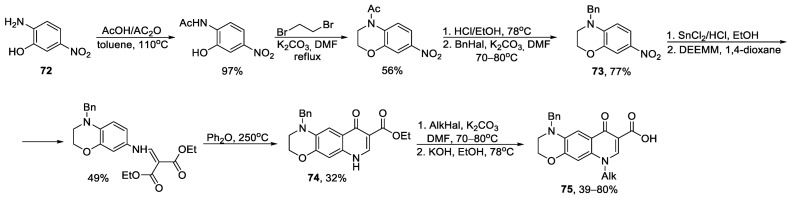
Synthesis of new tetrahydro-1H-[1,4]oxazino [3,2-g]quinoline derivatives.

In 2015, a method for the synthesis of new tetrahydro-1H-[1,4]oxazino [3,2-g]quinoline derivatives was patented [36]. The quite simple synthetic sequence starts from 2-amino-5-nitrophenol **72** as raw material and comprises successive N-acetylation, oxazine ring closure, and the introduction of benzyl group to give intermediate **73** (Scheme 16). Reduction of the nitro group and a further Gould–Jacobs reaction with diethyl ethoxymethylenemalonate afforded quinoline **74**, which on alkylation and ester hydrolysis yielded fused quinolines **75**. All compounds have a certain inhibitory effect on HIV-1 integrase with IC_50_ less than 500 nM.

**Scheme 17:**
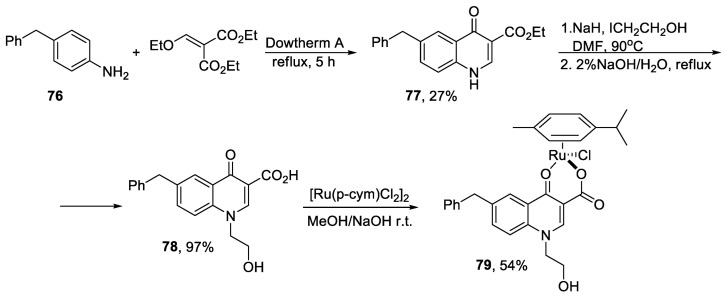
Synthesis of Ru(II) complex based on quinoline derivative as ligand—structural analog of Elvitegravir.

Carcelli et al. reported the first example of ruthenium complex based on quinoline derivative as a ligand—a structural analog of Elvitegravir [37]. Quinolone ligand was prepared from 4-benzylaniline **76** by heating with diethyl ethoxymethylenemalonate, subsequent thermal cyclization in Dowtherm A, *N*-alkylation, and hydrolysis of ether **77** to give compound **78** (Scheme 17) [38]. The ruthenium(II) complex **79** was synthesized by the reaction of **78** with [Ru(p-cym)Cl_2_]_2_ in MeOH. It was shown that both ligand and Ru-complex have inhibition potency in the micromolar concentration range.

**Scheme 18:**
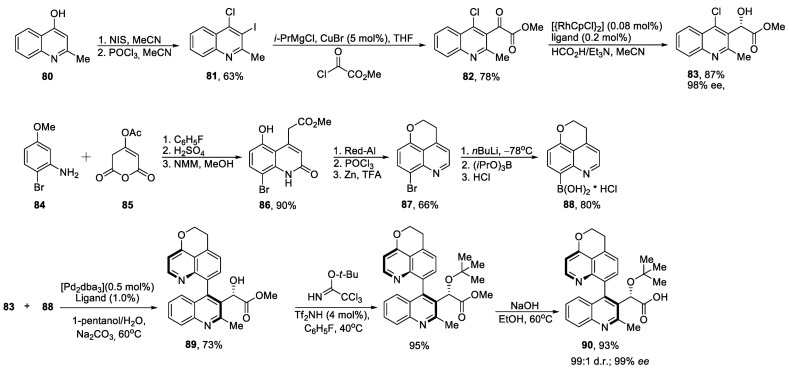
Fandrick’s efficient multi kilogram scale synthesis of quinoline based HIV-1 INI.

An efficient multi-kilo scale synthesis of quinoline based HIV-1 INI was accomplished by Fandrick et al. [39,40]. The molecule consists of two quinoline-based parts coupled together using Suzuki-Miyaura conditions (Scheme 18). For the synthesis of the first fragment, 4-hydroxy-2-methylquinoline **80** was used as starting compound. Its iodination with NIS and reaction with POCl_3_ gave iodide **81** in 63% overall yield. Acylation of **81** with methyl oxalyl chloride and asymmetric reduction of ketoester **82** provided compound **83** with excellent enantiopurity. The second structural unit was synthesized on the basis of 2-bromo-5-methoxyaniline **84**. It was acylated with unsaturated anhydride **85** and cyclized under the action of sulfuric acid to compound **86**. A further reaction sequence of reduction, chloro-dehydroxylation, and dechlorination afforded bromide **87**, which then was converted to the corresponding boronic acid **88** in 80% yield. Assembly of the target molecule skeleton was carried out by Suzuki–Miyaura cross-coupling of compounds **83** and **88**. Product **89** was *O*-tert-butylated and hydrolyzed to give compound **90** in 93% yield and high stereoselectivity. This protocol was patented in 2014 [41].

**Scheme 19:**
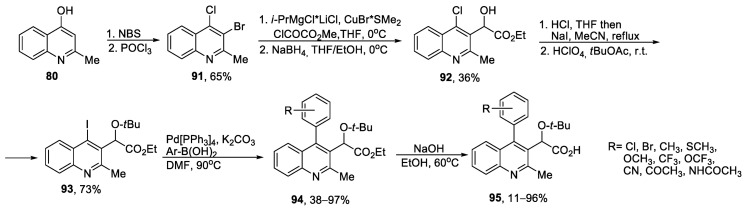
An approach to 4-arylquinoline derivatives **95**.

Jentsch et al. developed another library of 4-arylquinoline derivatives, which also possess activity against HIV-1 integrase (Scheme 19) [42]. The synthetic scheme is quite similar to the one mentioned above (Scheme 18), and it starts from commercially available 4-hydroxy-2-methyl-quinoline **80** that has undergone direct bromination and reaction with POCl_3_ to give intermediate **91** in 65% yield. It was then converted to α-hydroxyester **92** via acylation with methyl oxalyl chloride and reduction with NaBH_4_. Reaction of **92** with NaI followed by *tert*-butylation afforded 4-iodoquinoline **93**, which reacted with a number of arylboronic acids to give compounds **94**. Hydrolysis of esters **94** led to target carboxylic acids **95**. This study has provided relevant information regarding the structure–activity relationship and defining factors for candidate INIs design. 4-Chlorophenyl and 2,3-benzo[b][1,4]dioxine derivatives showed the highest potency against HIV-1 integrase.

**Scheme 20:**
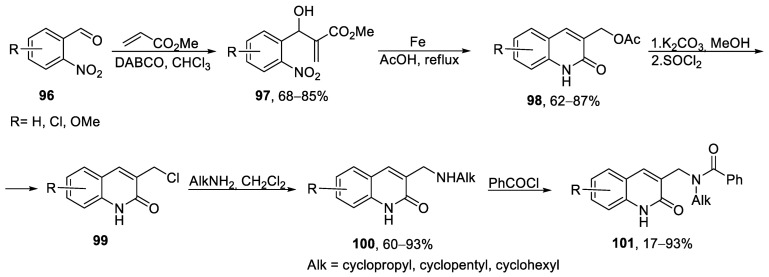
2-Quinolone-derived INIs.

A six-step method for the synthesis of 2-quinolone derivatives using Morita–Baylis-Hillman methodology was developed by Sekgota et al. [43]. Substituted 2-nitrobenzaldehydes **96** reacted with methyl acrylate in the presence of DABCO to give MBH adducts **97** followed by reductive cyclization to quinolones **98** (Scheme 20). They, in turn, were converted to 3-chloromethyl compounds **99** and then to secondary amines **100**. Acylation with benzoyl chloride provided amides **101**.

**Scheme 21:**
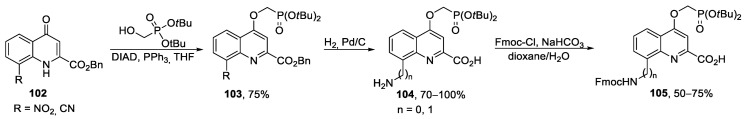
Synthesis of quinoline-based INIs bearing phosphonate groups.

Aromatic foldamers containing quinoline skeleton were proposed as HIV-1 INIs in 2018 [44]. The authors described the synthesis of several quinoline derivatives bearing phosphonate groups (Scheme 21). Phosphonate fragment was introduced into compounds **102** under Mitsunobu conditions and subsequent hydrogenation of 8-nitro- or 8-cyano derivatives **103** was accompanied with benzyl ester cleavage to give 8-amino- or 8-aminomethylquinolines **104,** respectively. At the last step, Fmoc protective group was installed, affording the corresponding amides **105** in moderate yields.

**Scheme 22:**
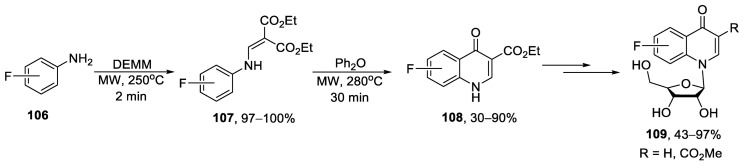
Synthesis of fluoroquinolone ribonucleosides **109**.

In 2006 a microwave-assisted synthesis of fluoroquinolone ribonucleosides was described [45]. Reaction of fluoroanilines **106** with diethyl (ethoxymethylidene)malonate followed by ring closure gave fluoroquinolone derivatives **108** (Scheme 22). The target compounds **109** were obtained by coupling with 1,2,3,5-*O*-tetraacetyl-D-ribofuranose and deprotection of hydroxyl groups with NaOMe in MeOH. All fluoroquinolones were examined as HIV-1 INIs. Interestingly, synthesized nucleosides were found to be inactive in cell culture but demonstrated enzymatic inhibitory effect against HIV-1 integrase.

**Scheme 23:**
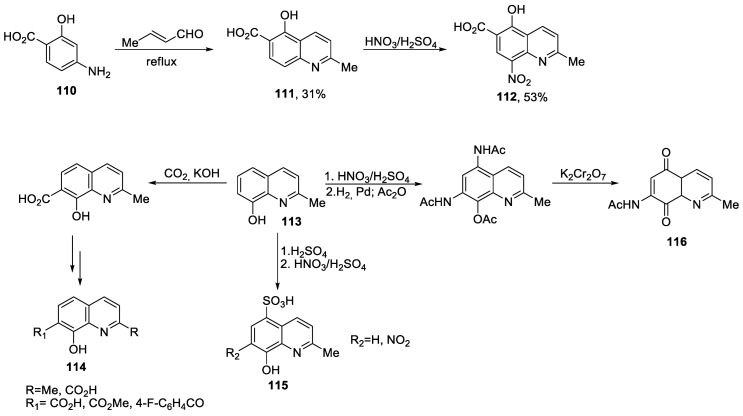
Polyfunctional quinaldines—potential HIV-1 INIs.

A number of simple quinaldines and their derivatives were synthesized as novel promising small molecular scaffolds—potential INIs [46,47]. Some examples are depicted on Scheme 23. Scraup reaction of 4-aminosalycilic acid **110** with crotonaldehyde gave quinoline **111**, which showed IC_50_ of 47 µM. Nitration of **111** afforded quinaldic acid **112** (IC_50_ 42 µM). Another synthetic route leads to target compounds starting from 8-hydroxyquinaldine **113**. This compound underwent various electrophilic functionalizations (nitration, carboxylation, or sulfonation) to give desired quinolines **114**–**116**, which were found to be less active than **112**. 

**Scheme 24:**
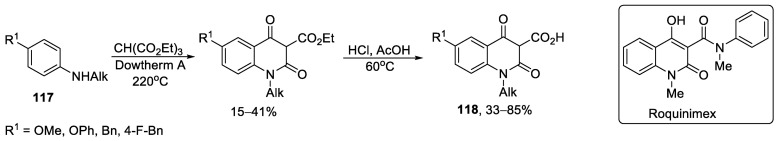
Synthesis of diketo-bioisosteric analogs of Roquinimex.

Dihydroquinoline-3-carboxylic acids were designed and synthesized by Sechi et al. (Scheme 24) [48]. Heating of corresponding N-alkyl anilines **117** with triethyl methanetricarboxylate in Dowtherm A followed by hydrolysis led to target carboxylic acids **118** in moderate yields. These compounds were designed as diketo-bioisosteric analogs of Roquinimex that inhibited both 3′-processing and strand transfer activities. The authors assumed that derivatization of Roquinimex by changing of amide for diketo fragment would improve inhibition effect. However, all synthesized derivatives showed potency in inhibiting of HIV-1 integrase at a level of Roquinimex or less along with low cytotoxicity.

**Scheme 25:**
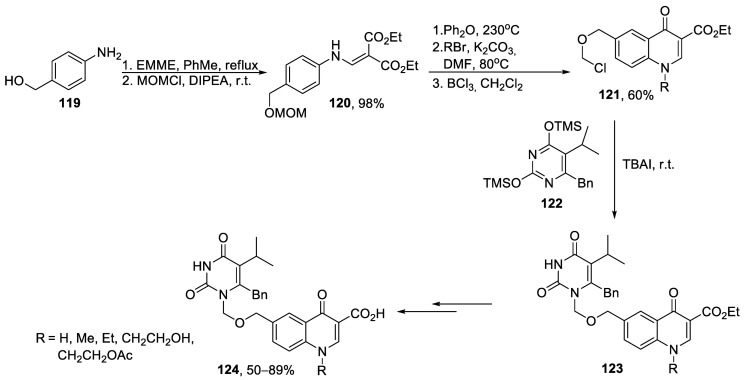
Wang’s synthesis of quinoline-pyrimidine hybrids **124**.

Wang et al. reported design and synthesis of quinoline-pyrimidine hybrids as potential dual inhibitors of HIV-1 integrase and reverse transcriptase [49]. These compounds represent Elvitegravir analogues with formal substitution of aryl fragment with pyrimidine scaffold. Quinoline moiety was assembled using Gould–Jacobs method (Scheme 25). 4-Hydroxymethylaniline **119** reacted with ethoxymethylenemalonic ester to give corresponding enamine, and MOM protection of the hydroxyl group gave compound **120**. Next, heating in Ph_2_O provided quinoline-4-one derivative, which on N-alkylation and protective groups interconversion gave chloromethyl derivative **121**. Pyrimidine fragment was then introduced by reaction with bis-(trimethylsilyloxy) compound **122**. The synthesized compounds demonstrated both anti-RT activity and anti-IN activity. In particular, the effect of inhibition of HIV-1 integrase was found in submicromolar concentrations (IC_50_ 0.19–3.7 µM).

**Scheme 26:**
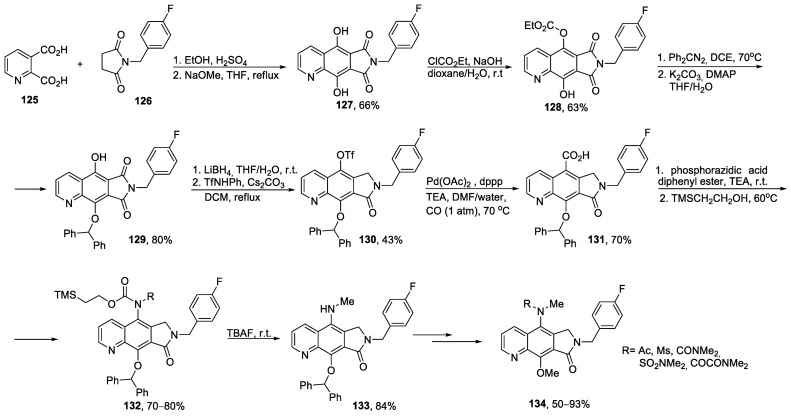
Synthesis of 6,7-dihydro-8H-pyrrolo[3,4-g]quinolin-8-one derivatives.

Approaches to the synthesis of tricyclic pyridine derivatives as potential HIV-1 INIs were reported by Kim et al. [50,51,52]. The authors synthesized several series of 7-(4-fluorobenzyl)-6,7-dihydro-8H-pyrrolo [3,4-g]quinolin-8-one derivatives and studied their inhibitory anti-HIV-1 activity. This class has shown great potential in integrase inhibition. One of the standard synthetic sequences leading to the target compounds is depicted in Scheme 26. Heterocyclic core was assembled by esterification of starting pyridine-2,3-dicarboxylic acid **125** followed by the Dieckmann condensation with 1-(4-fluoro-benzyl)-pyrrolidine-2,5-dione **126** with the formation of tricyclic *bis*-phenol **127**. One of the hydroxyl groups was then protected by reaction with ethyl chloroformate to give **128**, and another one was converted to (diphenylmethyl)oxy group (compound **129**). Reduction of one of the carbonyl groups and further conversion of OH to triflate afforded compound **130**, which was, in turn, transformed into carboxylic acid **131** and finally to methylamino derivative **133**. After several transformations, the desired tricyclic amides and sulfonamides **134** were obtained in moderate to high yields.

**Scheme 27:**
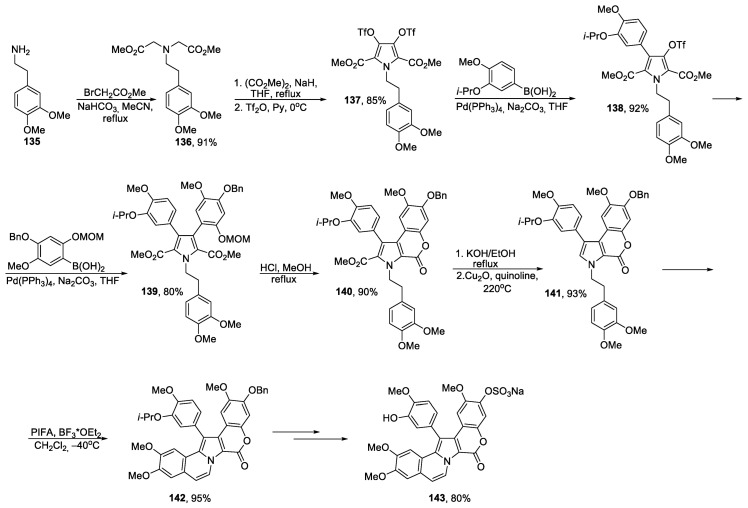
The first total synthesis of lamellarin α 20-sulfate.

In 2006, the first total synthesis of lamellarin α 20-sulfate as selective HIV-1 INI was reported [53]. Synthetic route started from commercially available 3,4-dimethoxyphenetylamine **135**, which was alkylated with bromoacetic ester to give the corresponding iminodiacetate **136** (Scheme 27). In the next step, the Hinsberg reaction with dimethyl oxalate followed by reaction with Tf_2_O led to bistriflate derivative **137**, which was successively arylated with two different boronic acids in the presence of Pd(PPh_3_)_4_ to give 3,4-diarylpyrrole **139**, which was then converted to six-membered lactone **140** and then decarboxylated to compound **141**. The intramolecular oxidative biaryl coupling under Kita’s conditions provided 20-benzyl-13-isopropyllamellarin a **142**. Finally, deprotection of the benzyl group, selective removal of the isopropyl protecting group, and deprotection of the trichloroethyl ester gave rise to target lamellarin a 20-sulfate **143**.

**Scheme 28:**
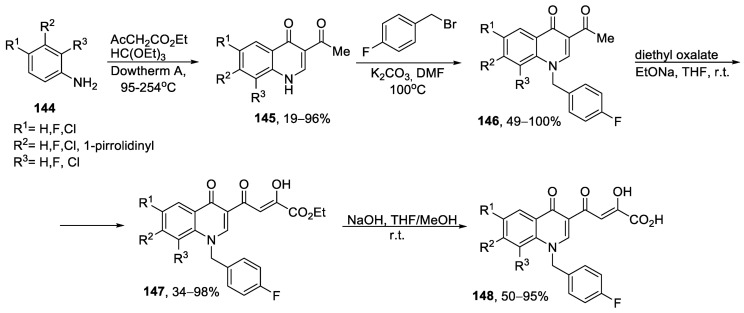
Synthesis of quinoline containing diketo acids **148**.

Novel quinoline containing diketo acids were designed by Di Santo et al. [54]. Commercial anilines **144** reacted with ethyl orthoformate and ethyl acetoacetate in Dowtherm A to give corresponding 3-acetyl-4(1*H*)-quinolinones **145** (Scheme 28). Compounds **145** were then N-alkylated with 4-fluorobenzylbromide followed by condensation with diethyl oxalate affording esters **147**, which were hydrolyzed in basic media to give the corresponding acids **148**. 8-Fluoroquinoline (**148**, R^3^ = F) was the most potent derivative in IN enzyme assays, 8-pyrrolidin-1-yl compound and showed the highest potency against HIV-1 in acutely infected cells.

**Scheme 29:**
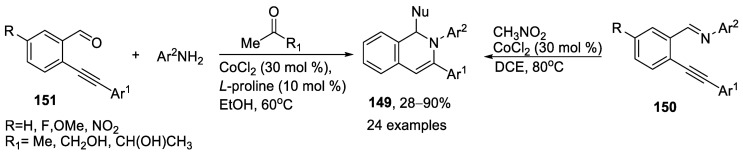
Synthesis of N-functionalized 1,2-dihydroisoquinolines.

Tandon et al. designed a series of C-1, C-3, C-7, and N-functionalized 1,2-dihydroisoquinolines and studied them as HIV-1 INIs [55]. The target compounds **149** were synthesized by Mannich condensation of *o*-alkynylaldimines **150** or by the reaction of o-alkynyl aldehydes **151** with ketones and amines (Scheme 29). All reactions proceeded under cobalt (II) chloride catalysis. Screening of these compounds revealed significant inhibition against strand transfer processes of HIV-1 and significant antiviral activity, which is comparable to Raltegravir.

**Scheme 30:**
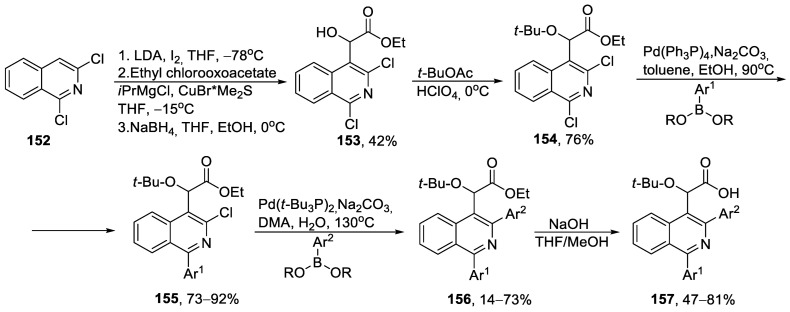
Synthesis of 1,3-dichloroisoquinoline derivatives as potential HIV-1 inhibitors.

An effort to expand of scope of potential HIV-1 inhibitors was done by Tyler et al. [56]. It was realized on the basis of the commercially available 1,3-dichloroisoquinoline **152** (Scheme 30). At the first step, the introduction of hydroxyacetic acid side chain into isoquinoline core was realized. Compound **153** was then converted into *t*-butyl ether **154** under the action of *t*-butyl acetate and HClO_4_. The authors found reaction conditions allowing to introduce two different aryl substituents in positions 1 and 3 of isoquinoline core. Thus, the first Suzuki–Miyaura coupling reaction with aryl boronic acid was carried out at 90 °C at more reactive position 1, while the second coupling with another boronic acid occurred under more drastic conditions to give 1,3-diarylisoquinoline esters **156**. Finally, hydrolysis of esters **156** afforded desired carboxylic acids **157**.

**Scheme 31:**
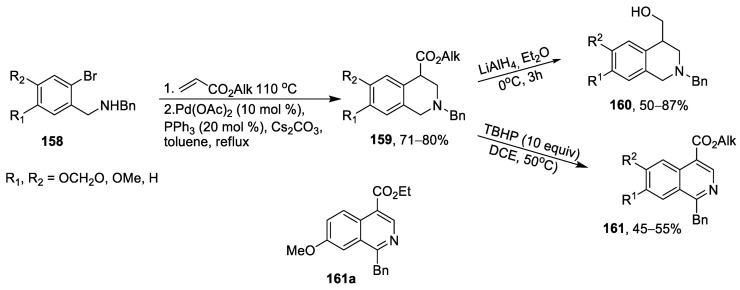
Synthesis of 1,2,3,4-tetrahydroisoquinoline and isoquinoline derivatives.

Satyanarayana et al. proposed a simple and efficient protocol for the synthesis of 1,2,3,4-tetrahydroisoquinoline derivatives [57]. The method is based on one-pot sequential intermolecular aza-Michael addition and Pd-catalyzed intramolecular Buchwald-Hartwig arylation of *bis*-benzylamines **158** (Scheme 31). Later, the same authors described chemical transformations of isoquinoline derivatives **159**, such as reduction to alcohols **160** and rearranged aromatization to give isoquinolines **161** [58]. Compounds **160** and **161** possessed higher anti-HIV activity compared to **159**. Compound **161a** was found to be the most effective among all other synthesized analogs. It efficiently inhibits the interaction between LEDGF/p75 and integrase in vitro, as well as HIV-1 infection in a cell line. Therefore, this compound has been identified as a lead molecule in this study.

**Scheme 32:**
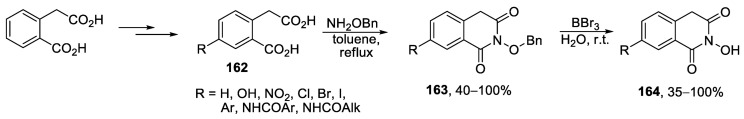
Design and synthesis of N-hydroxyisoquinoline derivatives.

A series of N-hydroxyisoquinoline derivatives was designed as potential dual inhibitors of HIV-1 integrase and reverse transcriptase RNase H domain by Billamboz et al. [59]. The method is based on the condensation of 7-substituted homophthalic acid derivatives **162** with O-benzylhydroxylamine to give the corresponding 2-benzy-loxyisoquinolines **163** followed by deprotection of the *N*-hydroxyl function yielding compounds **164** (Scheme 32). Most compounds inhibited RNase H and integrase at micromolar concentrations, and some of them were weakly selective for integrase.

**Scheme 33:**
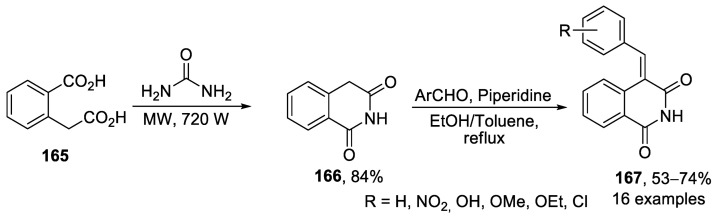
Synthesis of benzylideneisoquinoline-1,3(2*H*,4*H*)-diones **167**.

Synthesis of structurally similar 4-substituted benzylideneisoquinoline-1,3(2*H*,4*H*)-dione derivatives was published by Wadhwa [60]. Isoquinoline core was synthesized by the reaction of homophthalic acid **165** and urea under microwave irradiation (Scheme 33). Then isoquinoline-1,3(2*H*,4*H*)-dione **166** reacted with various aromatic aldehydes to give desired molecules **167**. Isoquinolines **167** were tested for anti-HIV-1 activity, and some of them exhibited significant percentage inhibition of HIV-1 integrase with IC_50_ values less than 5.96 μM.

**Scheme 34:**
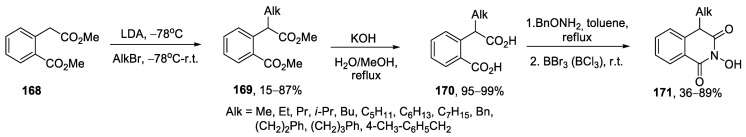
Synthesis of 2-hydroxyisoquinoline-1,3(2*H*,4*H*)-dione derivatives.

Another series of 2-hydroxyisoquinoline-1,3(2*H*,4*H*)-dione derivatives were synthesized [61]. All compounds were prepared from dimethyl homophthalate **168** in four steps (Scheme 34). Firstly, alkylation of the starting compound provided a number of monoalkyl derivatives **169**, which were hydrolyzed to the corresponding acids **170**. Isoquinoline ring closure was implemented via reaction with *O*-benzyl hydroxylamine, and deprotection under the action of boron trihalogenides gave desired N-hydroxyisoquinolines **171**. Some of the synthesized compounds inhibited HIV-1 integrase at a low micromolar level, although high cytotoxicity in cell culture limited their applications as antiviral agents.

### 2.3. Pyridines Fused with Heterocycles

Pyrazine-fused 3-hydroxypyridine-4-one moiety is a central part of the approved HIV-1 INIs Dolutegravir, Bictegravir, and Cabotegravir. A number of publications during the two prior decades has dealt with the design and synthesis of novel INIs of condensed piridinone series as well as of more efficient and cost-friendly procedures for known INIs [62]. For example, a process for the preparation of Dolutegravir and its sodium derivatives was reported in 2016 in the patent literature [63]. Seven-step flow synthesis of Dolutegravir was described, allowing the production of this pharmaceutical in sequential flow operations from commercially available materials [64]. The synthesis included rapid manufacturing time and the combination of multiple steps in order to avoid isolation of intermediates.

**Scheme 35:**
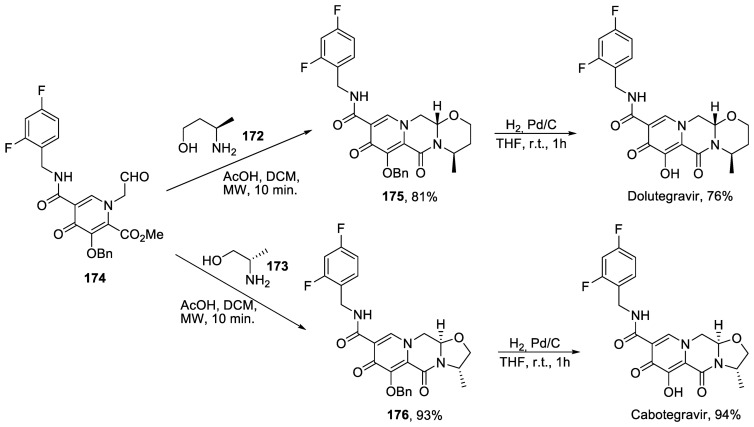
Divergent synthesis of Dolutegravir and Cabotegravir.

Most of the published synthetic approaches to Dolutegravir follow a similar strategy—construction of a pyridinone cycle and further annulation of saturated pyrazine and oxazine rings [65,66]. For example, synthesis of Dolutegravir, Cabotegravir, and a series of related compounds was described in 2015 [67]. Diastereoselective annulation of saturated heterocyclic rings to a pyridinone core was the main challenge of the research. For this reason, readily available chiral amino alcohols **172** and **173** reacted with previously synthesized aldehyde **174** [68] for hemiaminal ring fusion, furnishing the desired stereocenter within the tricyclic carbamoyl pyridinone scaffold (compounds **175** and **176**). The target compounds were obtained after cleavage of O-benzyl protection using hydrogen and Pd/C (Scheme 35).

**Scheme 36:**
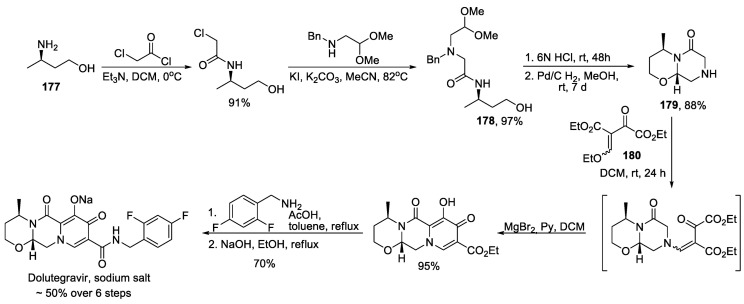
Alternative approach to Dolutegravir and its sodium salt.

Another approach was reported recently by Opatz et al. [69]. The authors proposed short and practical gram-scale synthesis of Dolutegravir sodium salt where assembling of the target molecule commenced with the formation of oxazine and pyrazine rings (Scheme 36). Chloroacetyl chloride as bis-electrophilic reagent first reacted with (3*R*)-amino-1-butanol **177** followed by reaction with (2,2-dimethoxyethyl)benzylamine. The resulting compound **178** was then cyclized and the benzylic protection cleaved to give rings B and C of Dolutegravir (compound **179**). Annulation of a pyridine ring was accomplished by the condensation with diethyl-2-(ethoxymethylidene)-3-oxobutandioate **180** and finally 2,4-difluorobenzylamine fragment was introduced and sodium salt was obtained on heating with ethanolic NaOH.

**Scheme 37:**
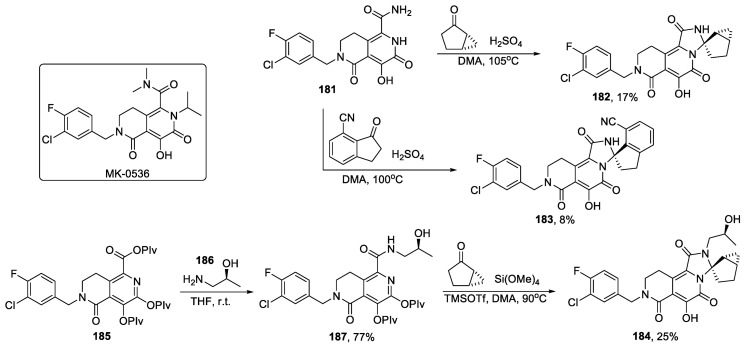
Synthesis of novel 2-pyridinone aminal series.

Novel 2-pyridinone aminal series was designed as potential INIs [70]. The previously reported clinical candidate MK-0536 served as starting point in the optimization process (Scheme 37). Reactions of substituted 2,6-naphthyridine-2-carboxamides **181** with cyclic ketones resulted in the formation of spiro aminals **182** and **183** in low yields. Another promising spirocompounds **184** was synthesized on the basis of pivaloyl-protected ester **185** by the reaction with chiral amine **186** to give amide **187** followed by reaction with bicyclic ketone. As a result of this work, several lead molecules were discovered and their structures were optimized in order to reach the optimum ratio of the antiviral activity values and physiochemical properties.

**Scheme 38:**
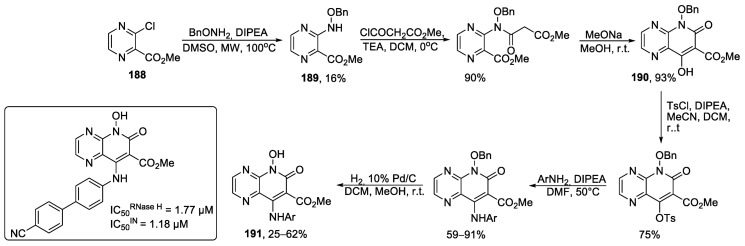
Synthesis of dual HIV-1 reverse transcriptase RNase H domain and integrase inhibitors.

The first dual HIV-1 reverse transcriptase RNase H domain and integrase inhibitors based on a 5-hydroxypyrido [2,3-*b*]pyrazin-6(5*H*)-one structure was reported in 2018 [71]. The synthetic route included the reaction of commercially available methyl 3-chloropyrazine-2-carboxylate **188** with *O*-benzyl hydroxyl amine leading to compound **189**, further acylation with methyl 3-chloro-3-oxopropanoate, and Claisen-type condensation to yield a bicyclic intermediate **190** (Scheme 38). It was then tosylated and reacted with aromatic amines of biphenyl and naphthalene series, followed by de-benzylation to give target compounds **191**. The majority of synthesized compounds inhibited both enzymes at micromolar concentrations. The best dual inhibitor possessed close IC_50_ values of 1.77 μM and 1.18 μM.

**Scheme 39:**
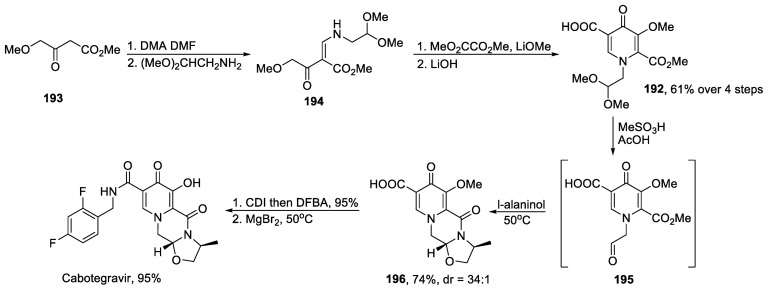
Wang’s diastereoselective synthesis of Cabotegravir.

An efficient and highly diastereoselective synthesis of Cabotegravir was reported by Wang et al. [72]. The method features a simple and efficient assembly of highly substituted pyridine-4-one core as well as diastereoselective construction of oxazolidine fragment. The intermediate pyridine-4-one **192** was obtained in 61% yield in a four-step one-pot operation, starting from methyl 4-methoxy-3-oxobutanoate **193**, which was treated with DMA DMF and then with 2,2-dimethoxyethylamine to give the corresponding enamine **194** (Scheme 39). This enamine underwent cyclization with dimethyl oxalate, and the addition of LiOH allowed the selective hydrolyzation of one of the methoxycarbonyl groups, viz. C-5. Acetal deprotection led to in situ formation of the aldehyde **195**, which reacted with *l*-alaninol and resulted in diastereoselective cyclization with formation of compound **196** with one more stereocenter (dr = 34:1). Finally, formation of 2,4-difluorobenzylamide and demethylation with MgBr_2_ gave Cabotegravir. 

**Scheme 40:**
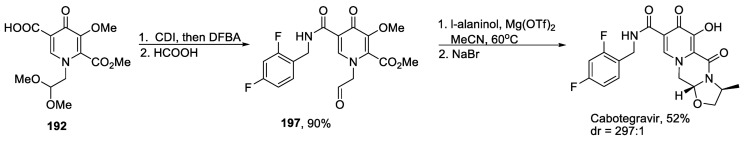
Diastereoselective synthesis of Cabotegravir.

Diastereomeric ratio in the final product was increased substantially by changing the steps order (Scheme 40). Formation of the amide followed by acetal deprotection resulted in the formation of aldehyde **197**, and reaction with *l*-alaninol in the presence of Mg(OTf)_2_ gave Cabotegravir with dr about 300:1.

**Scheme 41:**
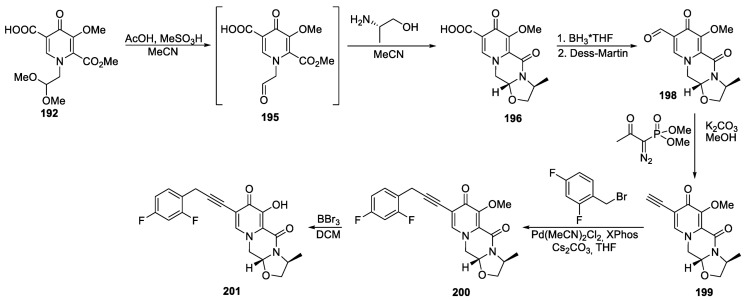
Synthesis of Cabotegravir analog **201**.

In addition to amide function, 2,4-difluorobenzyl fragment can be bound to the heterocyclic core through triple C-C-bond [73]. For this reason, the initial dimethyl acetal **192** was converted in situ to aldehyde **195** and then to tricyclic pyridinone carboxylic acid **196** (Scheme 41). Its reduction to alcohol followed by Dess–Martin oxidation gave the corresponding aldehyde **198**. Alkyne moiety was introduced by the reaction with Ohira–Bestmann reagent, and then terminal alkyne **199** was coupled with 2,4-difluorobenzyl bromide in Sonogashira reaction conditions. Demethylation of **200** with BBr_3_ afforded Cabotegravir **201** analog, which demonstrated significant activity (EC_50_ 67 nM) in an in vitro HIV pseudovirus.

**Scheme 42:**
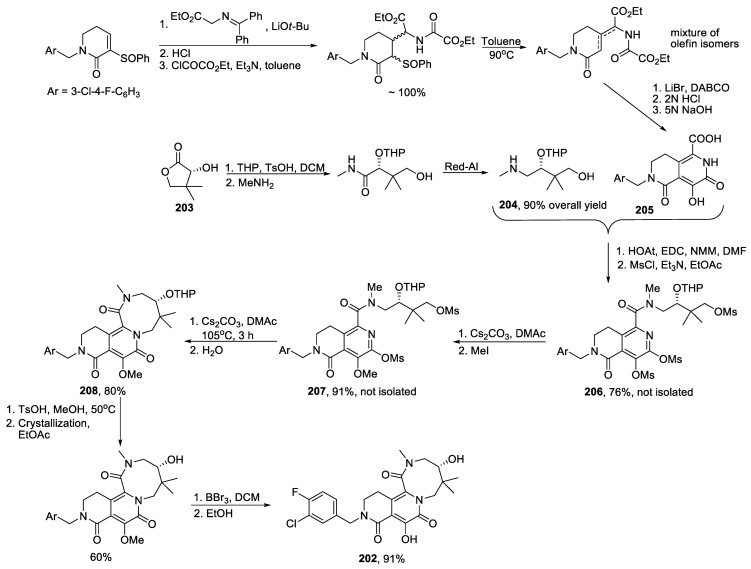
Practical asymmetric total synthesis of partially saturated naphthyridine **202**.

A practical asymmetric total synthesis of partially saturated naphthyridine **202** fused with 8-membered nitrogen heterocycle was developed [74]. Earlier, compound **202** was identified as a potent HIV-1 inhibitor [75]. The elaborated reaction sequence is depicted in Scheme 42 and includes more than 10 synthetic steps proceeding generally in good yields. The key steps are the formation of the naphthyridine system and the annulation of the eight-membered cycle. Readily available D-(−)-pantolactone **203** was taken as a precursor for the corresponding chiral amino alcohol **204**, which was used for the construction of the fused heterocyclic core via coupling with carboxylic acid **205**. Amide **206** without isolation was converted to tricyclic derivative **208** and after removal of THP and mesylate protective groups, the target compound **202** in 14% overall yield was obtained. It should be noted that the proposed approach did not require isolation of intermediates **206** and **207** during the formation of the 8-membered ring.

**Scheme 43:**
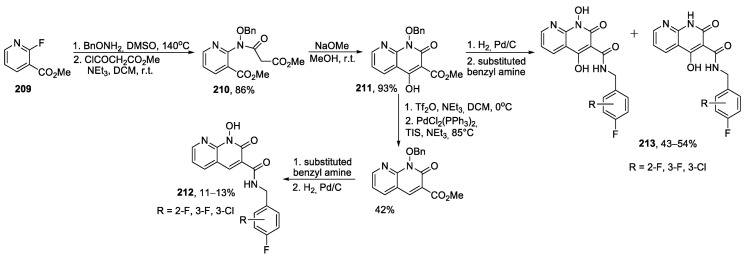
Preparation of bicyclic 2-oxo-1,2-dihydro-1,8-naphthyridine-3-carboxamides.

A series of bicyclic 2-oxo-1,2-dihydro-1,8-naphthyridine-3-carboxamides, highly potent against cells harboring Raltegravir-resistant integrase mutants, were synthesized and evaluated by Burke et al. [76]. Commercially available methyl 2-fluoronicotinate **209** was treated with *O*-benzyl hydroxylamine and further acylated with methyl 3-chloro-3-oxopropanoate to give compound **210** in 86% yield. Annulation of the second pyridine ring was achieved by Claisen-type condensation under the action of NaOMe. The resulting key intermediate **211** was used for the synthesis of both 1-hydroxy- and 1,4-dihydroxy-2-oxo-1,2-dihydro-1,8-naphthyridine-3-carboxamides **212** and **213,** respectively, as depicted in Scheme 43. Compounds bearing 2,4-difluorobenzylamide fragment demonstrated greater antiviral efficacy compared to that of RAL when tested against a panel of IN mutants, and thus represent potentially useful platforms for further structural variations in the search of more effective compounds.

**Scheme 44:**
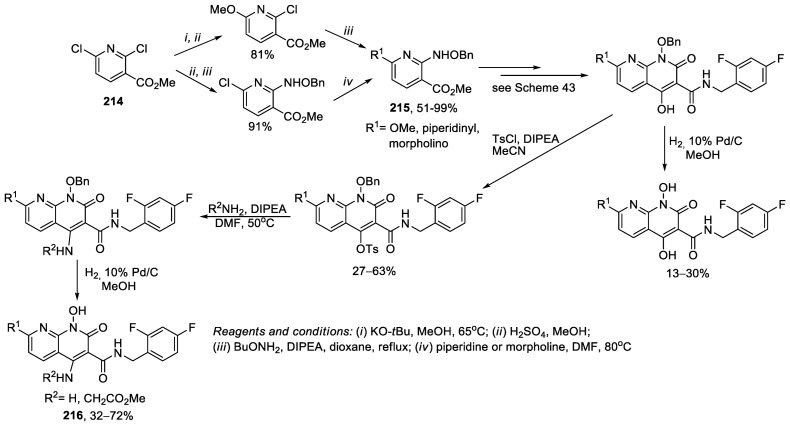
Synthesis of 1-hydroxy-2-oxo-1,2-dihydro-1,8-naphthyridine-3-carboxylic acid 2,4-difluorobenzylamides **216**.

Later, the same authors investigated the influence of substituents in position 6 and 7 of 1-hydroxy-2-oxo-1,2-dihydro-1,8-naphthyridine-3-carboxylic acid 2,4-difluorobenzylamides [77]. Basically, the synthetic scheme was quite similar to the one mentioned above, but starting compounds (nicotinic acid derivatives) bearing additional halogen atoms allowed the introduction of the necessary functions into the pyridine core. 2,6-Dichloronicotinic acid **214** was used as starting material in which both chlorine atoms were appropriately substituted to give compounds **215**. Annulation of another pyridine ring was carried out as shown in Scheme 43. Removal of benzyl group gave 7-substituted naphthyridines **216** (Scheme 44).

**Scheme 45:**
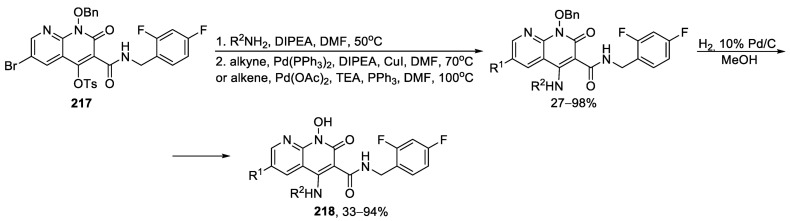
Synthesis of 6-substituted 2-oxo-1,2-dihydro-1,8-naphthyridine-3-carboxylic acid 2,4-difluorobenzylamides **218**.

The 6-Substituted compounds **218** were synthesized from 6-bromo derivatives **217** via Sonogashira or Heck reactions with alkynes or alkenes, respectively, followed by hydrogenation (Scheme 45).

**Scheme 46:**
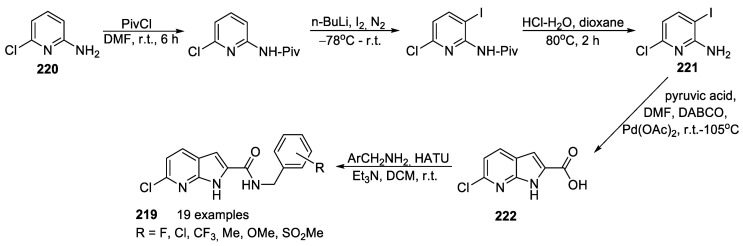
Synthesis of Pyrrolo[2,3-b]pyridine derivatives **219** as HIV-1 INIs.

Pyrrolo[2,3-b]pyridine derivatives **219** have been synthesized and considered as HIV-1 INIs [78]. Reaction of 2-amino-6-chloropyridine **220** with pivaloyl chloride followed by iodination and N-deprotection gave 2-amino-6-chloro-3-iodopyridine **221**, which was then reacted with pyruvic acid under Pd-catalysis resulting in pyrrole ring annulation to give compound **222** (Scheme 46). Pyrrolo[2,3-b]pyridine-2-carboxylic acid **222** thus obtained was converted to a variety of benzyl amides **219** on reactions with corresponding benzyl amine in the presence of HATU and Et_3_N. The yields were not indicated.

**Scheme 47:**
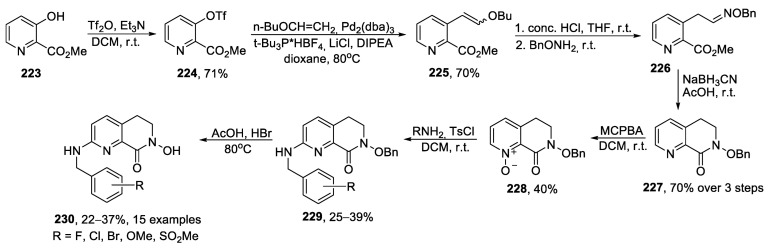
Synthesis of partially saturated 7-hydroxy-1,7-naphthyridines.

The same authors reported on the synthesis of partially saturated 7-hydroxy-1,7-naphthyridines having an inhibiting effect on HIV-1 integrase [79]. 3-Hydroxypicolinic acid **223** as a raw material was converted to triflate **224**, which then underwent Heck reaction with *n*-butyl vinyl ether (Scheme 47). Hydrolysis of the enol ether **225** and reaction with *O*-benzyl hydroxylamine gave corresponding oxime **226**, which on reduction with NaBH_3_CN gave cyclization product **227** in 79% yield. The introduction of benzylamine moiety in the pyridine cycle was realized via formation of N-oxide **228** under the action of MCPBA, followed by reaction with amine and TsCl. The final step deals with *O*-debenzylation of **229** in acidic media to give target compounds **230**. The most active compounds were found to be 4-F-, 4-Cl-, 3,4-F_2_-, 3,4-Cl_2_-, and 3-Cl-4-F-benzyl derivatives.

**Scheme 48:**
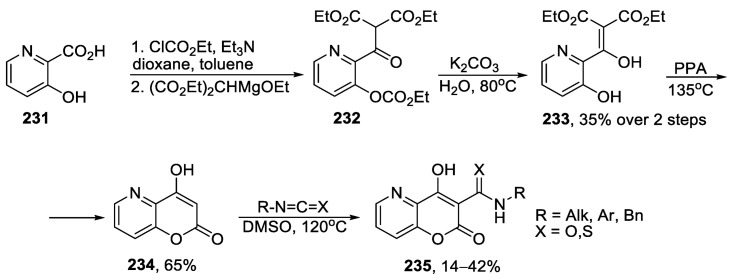
A method for the synthesis of new 4-hydroxy-5-azacoumarins.

A method for the synthesis of new 4-hydroxy-5-azacoumarin derivatives as potential HIV-1 inhibitors was proposed in 2007 [80]. 2-Hydroxypicolinic acid **231** reacted with ethyl chloroformate followed by condensation with ethyl ethoxymagnesium malonate to give compound **232** (Scheme 48). Partial hydrolysis under basic conditions gave enol **233**, which on heating in PPA underwent formation of azacoumarin **234**. Reactions of **234** with aryl isocyanates or aryl isothiocyanates led to the target 4-hydroxy-5-azacoumarin-3-carbox(thio)amides **235** in low to moderate yields. Carboxamides were found to be more active inhibitors compared to thioamides—IC_50_ 6.7–35.7 µM and >300 µM, respectively. Therefore, the 4-hydroxy-5-azacoumarin ring can be considered as a new scaffold in designing more potent HIV-1 INIs.

**Scheme 49:**
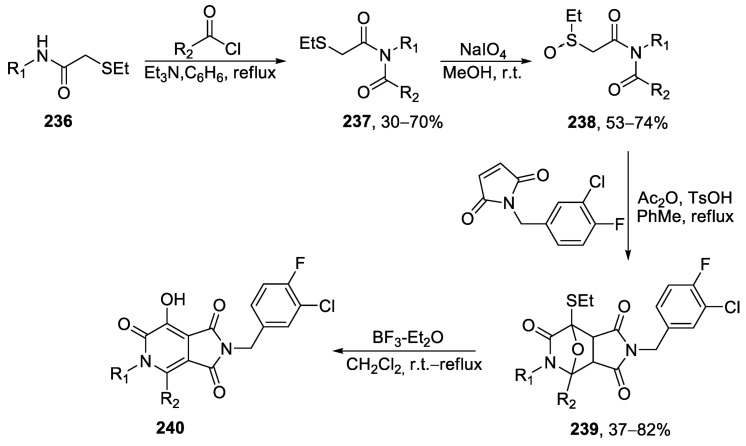
Zhao’s synthesis of novel bicyclic pyrrolopyridine-triones.

Zhao et al. reported on the synthesis of novel bicyclic pyrrolopyridine-triones, Scheme 49 [81]. The key step in synthesis is Pummerer-induced cyclization-deprotonation sequence. Acylation of (ethylthio)acetamides **236** with acyl chlorides afforded bis-acylamides **237**. The latter compounds were oxidized with sodium periodate to give sulfoxides **238** in good yields. Compounds were refluxed with N-(3-chloro-4-fluorobenzyl)maleimide giving rise to corresponding cycloadducts **239**, which on treatment with boron trifluoride diethyl etherate gave bicyclic pyrrolopyridines **240**. All compounds showed anti-HIV-1 activity with IC_50_ values in the range 6.4–21.6 µM.

**Scheme 50:**
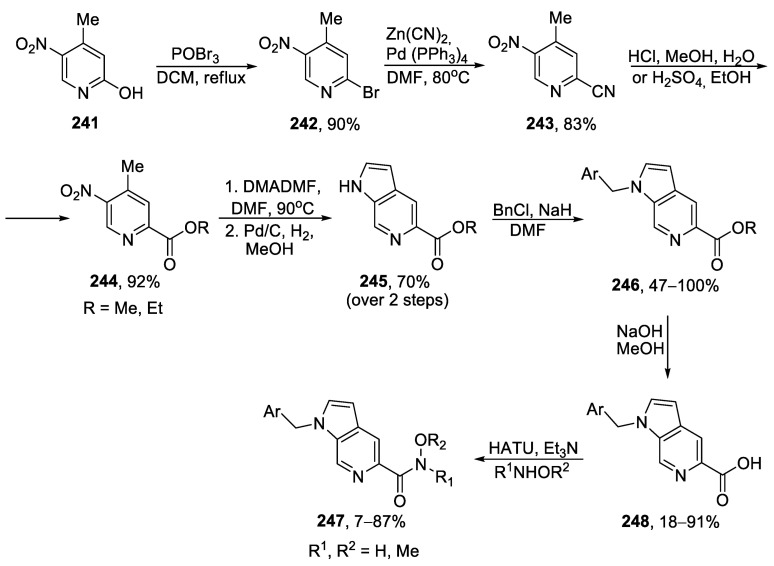
Synthesis of azaindole hydroxamic acids as potential HIV-1 INIs.

Plewe et al. studied azaindole hydroxamic acids as potential HIV-1 INIs [82]. They designed and proposed a method for the synthesis of 4-fluorobenzyl substituted azaindole hydroxamic acids (Scheme 50). Nitropyridine **241** was chosen as the starting material and reacted with POBr_3_ to give bromopyridine **242**. Bromide **242** was converted to nitrile **243** under the action of Zn(CN)_2_, and then to esters **244**. Reaction of **244** with DMADMF followed by hydrogenation led to azaindoles **245**. Synthesized esters were then alkylated with a number of benzyl halides, affording N-alkylazaindoles **246**. The latter compounds were transformed into hydroxamic acids **247**, either by direct reaction with hydroxyl amine or via hydrolysis to acids **248**, and further coupling with *N,O*-substituted hydroxyl amines.

**Scheme 51:**
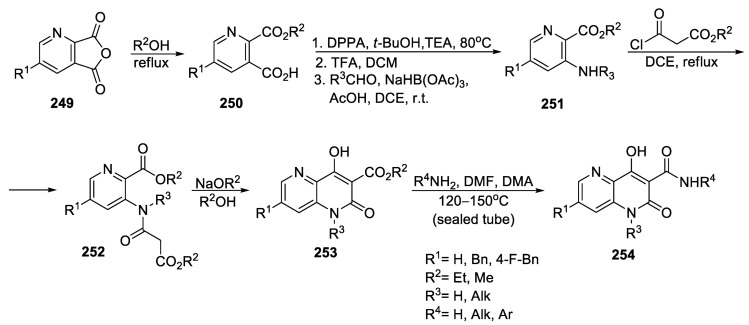
Synthesis of hydroxynaphthyridinone derivatives **254**.

A series of naphthyridinone derivatives were synthesized and their structure–activity relationship as HIV-1 INIs was disclosed by Johns et al., Scheme 51 [83,84]. Cyclic anhydrides **249** were ring-opened with alcohols to afford acids **250**. Curtius rearrangement of **250** under the action of DPPA in the presence of *t*BuOH gave N-Boc derivatives which were deprotected by TFA and underwent reactions with aldehydes and sodium triacetoxyborohydride leading to esters **251**. Reactions of **251** with methyl- or ethylmalonyl chloride produced intermediates **252** which were cyclized into naphthyridinones **253**. In turn, compounds **253** were converted into target carboxamides **254** by reactions with corresponding amines. The authors noted substituent effects at N-1, C-3, and 7-benzyl positions on inhibition activity.

**Scheme 52:**
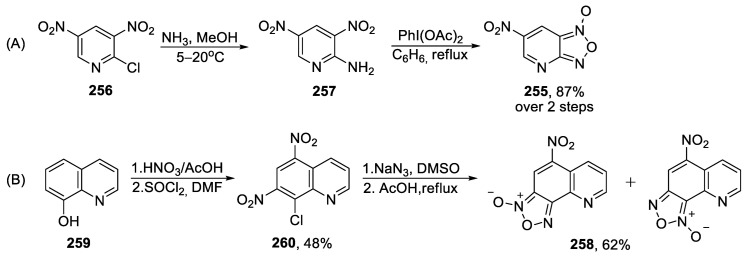
Synthesis of 4-aza-6-nitrobenzofuroxan **255** (**A**) and 5-nitro-7,8-furoxanoquinoline **258** (**B**).

Korolev et al. [85] showed that 4-aza-6-nitrobenzofuroxan **255** is able to inhibit two enzymes needed for successful HIV-1 replication: integrase and the RNase H domain within reverse transcriptase. Compound **255** was synthesized from commercially available 2-chloro-3,5-dinitropyridine **256** in two steps (Scheme 52A) [86]. Reaction of chloropyridine **256** with NaN_3_ followed by thermolysis of intermediate azide **257** afforded furoxanopyridine derivative in 87% overall yield. Structurally similar 5-nitro-7,8-furoxanoquinoline **258** also possesses anti-HIV activity. Its synthesis was described in [87,88] and started from 8-hydroxyquinoline **259** (Scheme 52B). Nitration of quinoline **259** and reaction with SOCl_2_ afforded chloride **260**. Nucleophilic substitution of its chlorine atom and further thermolysis of the intermediate o-nitroazide in boiling AcOH led to target furoxan **258** as a mixture of two regioisomers.

**Scheme 53:**
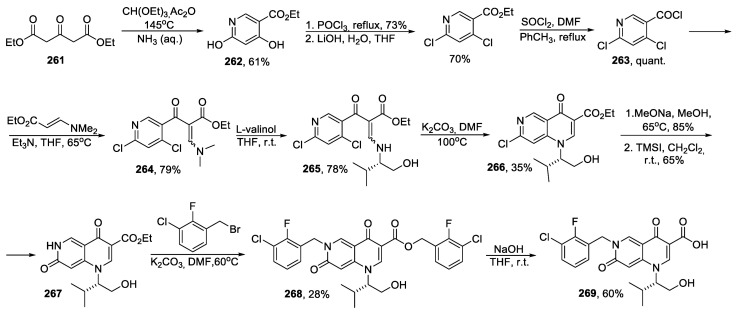
Synthesis of 1,6-naphthyridine-3-carboxylic acid **269**—structural analog of Elvitegravir.

Platts et al. developed a method for the synthesis of 1,6-naphthyridine-3-carboxylic acid—a structural analogue of Elvitegravir [89]. In the first step, commercially available diethyl 1,3-acetonedicarboxylate **261** reacted with triethyl orthoformate in acetic anhydride, followed by cyclization gave pyridine **262**, which was transformed into acid chloride **263** in three steps (Scheme 53). Compound **263** was treated with ethyl 3-dimethylaminoacrylate to give enamine **264**. Transamination of **264** with L-valinol afforded compound **265**, which, after cyclization, provided 1,6-naphthyridine derivative **266**. It was then converted to pyridine **267** and then treated with 2-chloro-3fluorobenzyl bromide with the formation of compound **268**. Hydrolysis of **268** under basic conditions led to Elvitegravir analogue **269** in good yield.

**Scheme 54:**
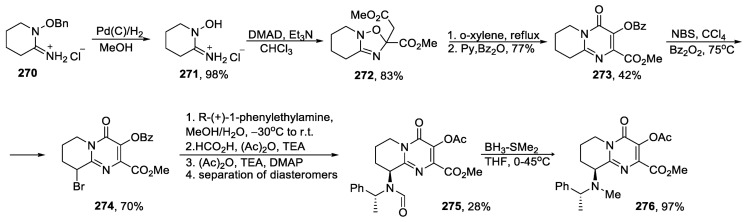
Synthesis of 6,7,8,9-tetrahydro-4*H*-pyrido[1,2-a]pyrimidines.

Synthesis of 6,7,8,9-tetrahydro-4*H*-pyrido[1,2-a]pyrimidines as an intermediate for a new class of HIV-1 INIs was reported by Kinzel et al. [90]. Cyclic amidoxime **270** was converted to 1-hydroxypiperidin-2-iminium chloride **271** via the cleavage of benzyl ether (Scheme 54). Compound **271** reacted with DMAD to afford 1,2,4- oxadiazoline **272**, which underwent rearrangement under reflux in o-xylene with the formation of pyrido[1,2-a]pyrimidine system **273**. After construction of bicyclic, the authors stereoselectively installed a chiral amino group in position 9 using a racemic bromide **274** as starting material. At the final step, N-formyl group in **275** was reduced to N-methyl using BH_3_SMe_2_ to give compound **276** in excellent yield.

**Scheme 55:**
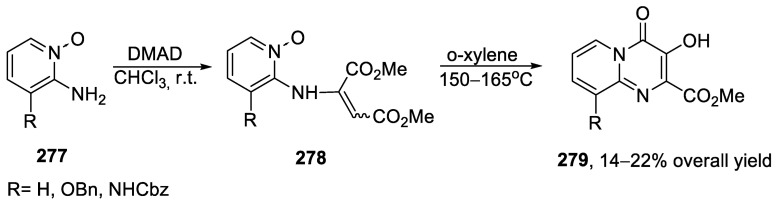
Synthesis of methyl-3-hydroxy-4-oxo-4*H*-pyrido-[1,2-a]pyrimidine-2-carboxylates **279**.

Later, in continuation of their research on HIV-1 INIs, the same authors reported the synthesis of new heterocyclic scaffold—methyl-3-hydroxy-4-oxo-4*H*-pyrido-[1,2-a]pyrimidine-2-carboxylates, Scheme 55 [91]. 2-Aminopyridine-N-oxides **277** readily reacted with DMAD, giving rise enamines **278** as mixture of E/Z-isomers. Heating of **278** in *o*-xylene afforded target pyrido[1,2-a]pyrimidines **279**.

**Scheme 56:**
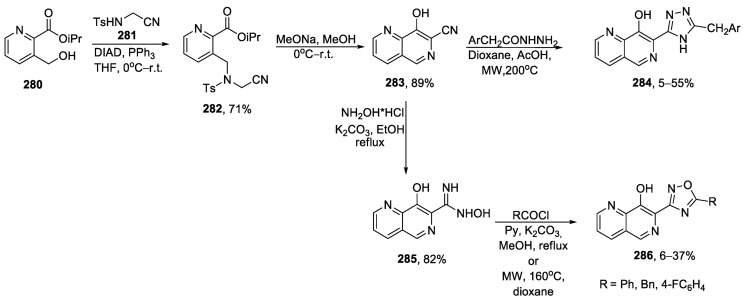
8-Hydroxy-1,6-naphtyridines conjugated with azoles.

Johns et al. described synthesis of 8-hydroxy-1,6-naphtyridines conjugated with azoles (oxadiazole or triazole) as HIV-1 INIs [92]. Mitsunobu reaction of compound **280** with tosylamide **281** followed by Dieckmann cyclization of the intermediate **282** afforded cyanonaphthyridine **283**, Scheme 56. Reaction of **283** with corresponding hydrazides under microwave conditions led to 1,2,4-trazole derivatives **284**. Another target hybrid system was 1,2,4-oxadiazole conjugated with naphtyridine core. The synthetic route for these compounds started from the reaction of nitrile **283** with hydroxylamine produced amidoxime **285**, which on treatment with acyl chlorides, either on heating in MeOH or under microwave conditions, provided target oxadiazoles **286**. As a result, the authors established the oxadiazole and the triazole heterocycles as viable components of the chelation motif involved in the 8-hydroxynaphthyridine integrase scaffold.

**Scheme 57:**
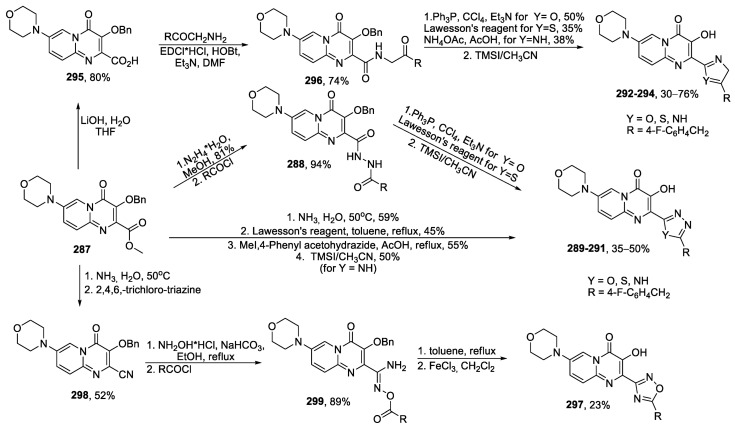
Synthesis of azole-containing pyrido[1,2-a]pyrimidines.

Synthesis of azole-containing pyrido[1,2-a]pyrimidines and their utilization as an amide isostere in HIV-1 integrase inhibition was reported by Le et al. [93]. Methyl ester **287** was chosen as a key starting material for the synthesis (Scheme 57). The ester group was converted mono-hydrazide, which was further acylated with 4-fluorophenylacetyl chloride to give intermediate **288**. Cyclization of this diacylhydrazide under the action of the corresponding agents and debenzylation with TMSI afforded 1,3,4-oxadiazole **289** and 1,3,4-thiadiazole **290**. Triazole **291** was synthesized directly from ester **287** by treatment of aqueous ammonium hydroxide followed by reaction with Lawesson’s reagent, and with MeI to give thioamide intermediate that cyclized with 4-F-phenyl acetohydrazide to form triazole ring. A similar strategy was used for synthesis of oxazole **292**, thiazole **293**, and imidazole **294**. Ester group in **287** was hydrolyzed to produce acid **295**, which underwent reaction with aminoketone affording **296**. During the next steps, cyclization and deprotection produce azoles **292**–**294**. Construction 1,2,4-oxadiazole derivative **297** was achieved via transformation of compound **287** into nitrile **298** by reaction with ammonia and dehydration with trichlorotriazine. Compound **298** was treated with hydroxylamine to give N-hydroxyamidine, which was acylated with 4-fluorophenylacetyl chloride to produce **299**, which on heating in toluene led to target oxadiazole **297**.

## 3. Conclusions

One of the goals of this review was to summarize most up-to-date approaches to the synthesis of known and new pyridine-based potential HIV-1 integrase inhibitors. An analysis of the literature over the last 20 years showed that the chemistry of monocyclic and fused pyridines is actively developing: syntheses of approved INIs are being improved and optimized, new syntheses are being published, and new pyridine-containing molecules are being created. Synthetic routes listed in this review are representative examples of the new developments, illustrating the importance of diverse pyridine compounds as feasible replacements or extensions of the field of anti-HIV agents.

Numerous studies confirm the importance of certain structural motifs to be included in a molecule to enhance activity, such as 4-fluoro-, 2,4-difluoro-, and 3-chloro-4-fluorobenzyl amides, 2,4-diketoacid fragment, or pyridin-4-one-3-carboxylic acid moiety in both monocyclic and condensed compounds. Since the structure of integrase is well established, including crystal and NMR studies of the individual domains, it can be predicted roughly if the designed compound would effectively block HIV replication. For this reason, molecular modeling methods, theoretical and computational, could help synthetic chemists in their routine research.

Inhibitory efficiencies are very important characteristics of the reviewed compounds. Indeed, IC_50_ values of the target molecules vary widely from nM (similar to approved compounds) to 100 mM concentrations which are unacceptable for use as INIs. In addition, some of them possess high toxicity, and the others have not been tested yet. Nevertheless, high IC_50_ and toxicity values do not automatically mean that the certain class of compounds should not be studied further. The main point is to find a new type of INIs that would allow to overcome resistance of the virus. Therefore, it is difficult to select the most promising compounds, and even the most promising class based solely on half maximal effective or inhibitory concentrations.

Thus, the field of chemistry associated with the synthesis of novel HIV-1 integrase inhibitors based on pyridine scaffold continues to be in demand and relevant. The results presented may serve as a basis for design and search of novel anti-HIV drugs.

## Data Availability

Not applicable.

## Abbreviations

AIDS	Acquired immunodeficiency syndrome
ARVT	Antiretroviral therapy
BIC	Bictegravir
BSA	Benzenesulfonic acid
CDI	1,1′-Carbonyldiimidazole
DABCO	1,4-Diazabicyclo[2.2.2]octane
DCE	1,2-Dichloroethane
DCM	Dichloromethane
DEEMM	Diethyl ethoxymethylenemalonate
DFBA	2,4-Difluorobenzyl amine
DIAD	Diisopropyl azodicarboxylate
DIPEA	N,N-Diisopropylethylamine
DMA	Dimethylacetamide
DMAD	Dimethyl acetylenedicarboxylate
DMADMF	Dimethylformamide dimethyl acetal
DMF	Dimethylformamide
DMSO	Dimethylsulfoxide
DPPP	1,3-Bis(diphenylphosphino)propane
DTG	Dolutegravir
EC_50_	Half maximal effective concentration
EDC (EDCI)	1-Ethyl-3-(3-dimethylaminopropyl)carbodiimide
EMME	Ethoxymethylenemalonic ester
EVG	Elvitegravir
FDA	Food and Drug Administration
FGI	Functional group interconversion
HATU	1-[Bis(dimethylamino)methylene]-1H-1,2,3-triazolo[4,5-b]pyridinium 3-oxide hexafluorophosphate
HIV	Human immunodeficiency virus
HOBt	Hydroxybenzotriazole
IC_50_	Half maximal inhibitory concentration
INI	Integrase inhibitor
MCPBA	Meta-chloroperoxybenzoic acid
NBS	N-Bromosuccinimide
NIS	N-Iodosuccinimide
NMM	N-Methylmorpholine
PIFA	Phenyliodine bis(trifluoroacetate)
PPA	Polyphosphoric acid
RAL	Raltegravir
TBHP	Tert-butyl hydroperoxide
TEA	Triethylamine
TFA	Trifluoroacetic acid
THF	Tetrahydrofuran
